# Endosperm and whole grain rye breads are characterized by low post-prandial insulin response and a beneficial blood glucose profile

**DOI:** 10.1186/1475-2891-8-42

**Published:** 2009-09-25

**Authors:** Liza AH Rosén, Lorena O Blanco Silva, Ulrika K Andersson, Cecilia Holm, Elin M Östman, Inger ME Björck

**Affiliations:** 1 Division of Applied Nutrition and Food Chemistry, Department of Food Technology, Engineering and Nutrition, Lund University, PO Box 124, SE-221 00 Lund, Sweden; 2 Division of Diabetes, Metabolism and Endocrinology, Department of Experimental Medical Sciences, Lund University, BMC F 10, SE-221 84 Lund, Sweden

## Abstract

**Background:**

Rye products have previously been shown to induce comparatively low post-prandial insulin responses; irrespectively of their glycaemic indices (GI). However, the mechanism behind this lowered insulin demand remains unknown. An improved insulin economy might contribute to the benefits seen in epidemiological studies with whole grain diets on metabolic risk factors and weight regulation. The objective of this study was to explore the mechanism for a reduced post-prandial insulin demand with rye products.

**Methods:**

12 healthy subjects were given flour based rye products made from endosperm, whole grain or bran, produced with different methods (baking, simulated sour-dough baking and boiling) as breakfasts in random order in a cross-over design. White wheat bread (WWB) was used as a reference. Blood glucose, serum insulin, plasma ghrelin and subjective satiety were measured during 180 minutes. To evaluate the course of post-meal glycaemia, a measure of the glycaemic profile (GP) was introduced defined as the duration for the incremental post-prandial blood glucose response divided with the blood glucose incremental peak (min/mM).

**Results:**

The study shows that whole grain rye breads and endosperm rye products induced significantly (p < 0.05) lower insulinaemic indices (II's) than WWB. Rye bran bread (RBB) produced significantly higher II compared with all the other rye products. Furthermore, the acute insulin response showed better correlations with the GP than with the GI of the products. The endosperm rye bread and the whole grain rye bread with lactic acid induced a significantly higher GP than RBB, WWB, white wheat- and whole grain rye porridge, respectively. A low insulin incremental peak was associated with less severe late post-prandial hypoglycaemia (r = 0.38, p < 0.001), and hypoglycaemia was negatively correlated to subjective satiety at 180 min (r = -0.28, p < 0.05). A low insulin incremental peak was also associated with a milder recovery of plasma ghrelin in the late post-prandial phase (180 min, r = 0.34, p < 0.01).

**Conclusion:**

Our study shows that endosperm and wholegrain rye products induce low acute insulinaemic responses and improved glycaemic profiles. The results also suggest that the rye products possess beneficial appetite regulating properties. Further studies are needed to identify the unknown property or bioactive component(s) responsible for these beneficial metabolic features of rye.

## Background

Whole grain products have been shown to protect against type 2 diabetes and CVD [1-7] and also to facilitate weight regulation [2,6,8,9]. Dietary fibre and other potentially bioactive compounds e.g. antioxidants, vitamins and minerals present in whole grains have been shown to contribute to the protective properties of whole grains foods [10-14]. However, the underlying mechanisms remain unclear. Foods with low glycaemic indices (GI) have also been demonstrated to protect against type 2 diabetes and CVD [1,15-18]. One mechanism behind the protective effect of low GI foods in relation to CVD may be the avoidance of frequent and elevated blood glucose excursions, which are associated with oxidative stress and inflammation [19]. Furthermore, a low GI is generally accompanied by a low acute insulin response. Consequently, hyperinsulinaemia lasting for 48-72 hours under physiologic euglycaemic conditions has been reported to decrease insulin sensitivity in healthy subjects [20]. In a recent dietary intervention in subjects suffering from the metabolic syndrome, it was shown that foods causing low acute insulinaemia may be less prone to promote sub-clinical inflammation [21], a feature commonly associated with insulin resistance [22,23].

Based on the above, whole grain foods characterized by a low GI/low insulineamia are of particular interest as type 2 diabetes and CVD preventing foods. Previous studies have demonstrated that whole grain rye products display low insulinaemic index (II), regardless of their GI [24-26]. This anomaly between the GI and II that is sometimes is present in whole grain rye products has not yet been explained and may indicate improved insulin economy. One suggested mechanism for the lowered acute insulinaemia with whole grain rye bread products include the structural features of rye bread; leading to obstructed amylolysis and a lowered rate of glucose delivery to the blood [25]. However, a lowered rate of glucose delivery caused by such a mechanism is likely to affect also the glycaemia to rye bread, and the insulin saving properties of certain whole grain rye products remain obscure.

The aim of the present study was to investigate the potential improvement of insulin economy following rye products, with a particular focus on evaluating the influence of different fractions of rye. For this purpose, rye products were produced from different parts of the rye grain (endosperm, whole grain and bran), and processed with different methods (baking vs. boiling). Since rye bread is commonly produced by use of sour-dough fermentation, the effect of lactic acid addition was also evaluated. The products were studied as to their effects on post-prandial glucose and insulin response in healthy subjects using white wheat bread as reference. In parallel, subjective satiety and total ghrelin responses were studied in the post-prandial phase. Additionally, the rate of in vitro starch hydrolysis was determined in order to evaluate the possibility of obstructed amylolysis.

## Methods

### Test subjects

Twelve healthy non smoking volunteers (9 men and 3 women) aged 25.3 ± 0.8 y with normal body mass indices: 23.1 ± 0.6 kg/m^2^, and without drug therapy participated in the study. All subjects had normal fasting blood glucose concentrations (4.6 ± 0.03 mM). The subjects were recruited in August 2005 and the study was performed from September to December 2005. All test subjects gave their informed consent and were aware of the possibility of withdrawing from the study at any time they desired. Approval of the study was obtained by the Ethics Committee in Lund, Sweden.

### Test meals

Four rye breads, two rye porridges, one white wheat (endosperm) porridge and white wheat (endosperm) bread (WWB, reference product) were included in the study. Whole grain rye flour, endosperm rye flour and rye bran from commercial blends were provided by Lantmännen R&D (Järna, Sweden) and commercial white wheat flour was obtained from Kungsörnen AB (Järna, Sweden). The rye bran was milled to pass through a 0.8 mm screen (Laboratory Mill 12, Perten, Huddinge, Sweden). Dry yeast was obtained from Jästbolaget AB (Sollentuna, Sweden) and lactic acid (88-92% extra pur) was obtained from Riedel-de Haën (Morris Township, NJ, USA). Monoglycerides were obtained from Aromatic (Stockholm, Sweden).

#### Breads

The ingredients of the breads are shown in Table 1. WWB was made in a bread machine (BM 3983, Severin, Sundern, Germany) using a program for white bread: The dough was mixed for 30 minutes and was proofed for 130 min, with 10 seconds short stirring every 39, 31 and 60 min. Baking was then performed for 55 minutes.

**Table 1:** Bread ingredients.

**WWB**	**ERB**	**WGRB**	**WGRB-lac**	**RBB**
360 g water	950 g water	1020 g water	995 g water	1100 g water
540 g white wheat flour	348 g white wheat flour	348 g white wheat flour	348 g white wheat flour	905 g white wheat flour
4.8 g dry yeast	1044 g endosperm rye flour	1044 g whole grain rye flour	1044 g whole grain rye flour	487 g rye bran flour
4.8 g NaCl	24 g dry yeast	24 g dry yeast	24 g dry yeast	24 g dry yeast
12 g monoglycerides	12 g NaCl	12 g NaCl	12 g NaCl	12 g NaCl
			25 g lactic acid	

The breads were divided into portions contributing with 40 g of available starch.

Four types of rye breads were made: Endosperm rye bread (ERB), whole grain rye bread (WGRB), whole grain rye bread with lactic acid (WGRB-lac) containing 18 mmol lactic acid/100 g flour and rye bran bread (RBB). All rye breads were made using a uniform method: The dough was mixed in a mixing bowl for 6 min and was proofed in room temperature for 30 min. The dough was divided into pieces of 1 kg each and placed in a bread making tin, followed by a second proofing for 60 min in room temperature. Baking was performed at 250°C for 40 min.

The WWB was left to cool for 1 hour and the rye breads for 18 hours under a cloth. Thereafter, the crust was removed and the breads were sliced and wrapped in aluminium foil in portions sizes, put into plastic bags and stored in a freezer (-18°C) until use. The day before the experiment, the breads were taken from the freezer and were thawed at ambient temperature, still wrapped in aluminium foil and in the plastic bag.

#### Porridges

Three types of porridges were cooked: white wheat porridge (WWP), endosperm rye porridge (ERP) and whole grain rye porridge (WGRP). The ingredients of the porridges are shown in Table 2. All porridges were cooked in a microwave oven (MM 140-1, Elektro Helios AB, Stockholm, Sweden) at 680 W for 3 min. The porridges were freshly prepared each experimental day and were left to cool under aluminium foil for 15 min before serving.

**Table 2:** Porridge ingredients.

**WWP**	**ERP**	**WGRP**
231.6 g water	182 g water	204.5 g water
57.9 g white wheat flour	15.2 g white wheat flour	17.0 g white wheat flour
0.5 g NaCl	45.4 g endosperm rye flour	51.1 g whole grain rye flour
	0.5 g NaCl	0.6 g NaCl

The porridges contributed with 40 g of available starch.

### Chemical analysis of the test products

Prior to all analyses, except for the determination of starch hydrolysis, the samples were dried and milled to pass through a 0.5 mm screen (Cyclotec, Tecator, Höganäs, Sweden).

The available starch content of the products was determined according to Holm et al. [27]. Insoluble and soluble fibres were determined with a gravimetric, enzymatic method described by Asp et al. [28]. Fat was determined according to Lange [29] with the exceptions that petroleum ether BP 60-80°C was used instead of petroleum ether BP 40-60°C. 10 ml of each ether was used instead of 15 in the second and third washing step. Protein content was determined by Kjeldahl analysis (Kjeltec Auto 1030 Analyser, Tecator, Höganäs, Sweden). The rate of starch hydrolysis (HI) was determined using an in vitro procedure based on chewing [30]. WWB was used as a reference in the HI analysis. The nutritional compositions and the HI values of the products are presented in Table 3.

**Table 3:** Composition and HI of the test meals.

**Meals**	**Weight**	**Available starch**	**Protein**	**Fat**	**Insoluble fibres**	**Soluble fibres**	**Total fibres**	**HI**
	*g/serving*							
WWB	101.1	40.0	6.2	1.5	1.0	0.8	1.8	100 ^a^
WWP	273.3	38.2	5.7	1.1	2.1	0.5	2.6	85 ± 4.4 ^bc^
ERB	106.2	40.0	5.2	1.3	4.2	2.5	6.7	83 ± 1.8 ^bc^
ERP	227.6	37.7	4.6	1.3	4.9	1.7	6.5	89 ± 3.6 ^b^
WGRB	123.4	40.0	6.5	1.9	6.8	2.8	9.6	101 ± 3.1 ^a^
WGRB-lac	122.6	40.0	6.3	2.0	7.4	2.9	10.2	94 ± 4.4 ^ab^
WGRP	258.2	38.6	5.4	1.7	7.9	2.2	10.1	72 ± 2.4 ^c^
RBB	141.7	40.0	9.7	2.6	10.3	2.0	12.3	93 ± 2.3 ^ab^

HI values are means ± SEM, n = 6. Products not sharing the same letters were significantly different, p < 0.05 (ANOVA, followed by Tukey's test).

### Study design

The products were provided as breakfasts on 8 different occasions in random order with approximately 1 wk between each test. The subjects were instructed to eat a standardized meal in the evening (21.00-22.00) prior to the test, consisting of a few slices of white wheat bread. They were instructed to avoid eating and drinking anything but small amounts of water until the start of the test. In addition, they were told to avoid alcohol and excessive physical exercise the day before each test. The subjects arrived at the laboratory at 07.45 on the test day. A peripheral venous catheter (BD Venflon, Becton Dickinson, Helsingborg, Sweden) was inserted into an antecubital vein to be used for blood sampling and fasting blood samples were taken prior to the meal. All test products contributed with 40 g of available starch and were served with 250 ml of tap water. The test subjects were instructed to finish the test product and water within 12 min. The subjects feeling of hunger and satiety was rated on a bipolar subjective rating scale graded from -10, representing extreme hunger, to + 10, representing extreme satiety. The feeling of hunger/satiety was rated before the meal (0 min) and at 15, 30, 45, 70, 95, 120 and 180 min after commencing breakfast. The test subjects were not allowed any further water or any caffeinated drinks during the test.

### Blood sampling and analysis

Both capillary and venous blood samples were taken at 0, 7.5, 15, 30, 45, 70, 95, 120 and 180 min after the start of the meal for analysis of blood glucose, serum insulin and plasma ghrelin. Blood glucose concentrations were determined in capillary whole blood using a B-glucose analyzer (mod no. 120401, Hemocue, Ängelholm, Sweden). Serum and plasma (EDTA) were left in room temperature for approximately 1 h before being centrifuged for 11 min (1800·g, 20°C). Serum were frozen at -20°C and plasma where frozen at -40°C until analysis. The serum insulin measurement was performed on an integrated immunoassay analyzer (CODA Open Microplate System; Bio-rad Laboratories, Hercules, CA, USA) by using an enzyme immunoassay kit (Mercodia AB, Uppsala, Sweden). Plasma ghrelin (total) were determined with a commercially available radioimmunoassay kit (Linco research inc, St. Charles, MO, USA).

### Calculations and statistical methods

Data are expressed as means ± SEM. The incremental areas under the curve (iAUC) for blood glucose, serum insulin, subjective satiety and in vitro rate of starch hydrolysis as well as the negative area under the curve for glucose (neg AUC), were calculated using the trapezoid model. The glycaemic and insulinaemic indexes (GI and II) were calculated from the 120 min incremental post-prandial area for blood glucose and serum insulin by using WWB as a reference (GI and II = 100). In addition, the course of glycaemia was analyzed by calculation a glycaemic profile (GP); The time (min) during which the blood glucose was above fasting concentration was divided with the incremental peak value (mM) of blood glucose for each subject and test meal (Graph Pad Prism, version 4.03, Graph Pad Software, San Diego, CA, USA). In the cases where the blood glucose concentration remained above fasting for the entire 180 min, the duration value was set to 180 min. A GP index was calculated from the GP by using WWB as a reference (GP Index = 100). HI was calculated from the 180 min incremental area for starch hydrolysis in vitro by using WWB as a reference. Relative changes (%) from fasting concentration to the nadir and to the concentration at 180 min after commencing breakfast were calculated for plasma ghrelin.

The data were analyzed with a general linear model (ANOVA) followed by Tukey's multiple comparison test (MINITAB, release 14.13, Minitab Inc, State College, PA). In the cases of unevenly distributed residuals (tested with Anderson-Darling and considered unevenly distributed when p < 0.05), Box Cox transformation were performed on the data prior to the ANOVA.

Significant difference between the products at different time points where evaluated using a mixed model (PROC MIXED in SAS release 8.01, SAS Institute Inc, Cary, NC) with repeated measures and an autoregressive covariance structure. When significant interactions between treatment and time were found, Tukey's multiple comparison test were performed for each time point (MINITAB, release 14.13, Minitab Inc).

Correlation analysis was conducted to evaluate the relation among dependent measures with the use of Spearman's partial coefficients controlling for subjects (two-tailed test), (SPSS software, version 16.0; SPSS Inc, Chicago, IL, USA).

Due to problems drawing capillary blood samples from one subject, the blood glucose statistics was analyzed with n = 11. One subject failed to ingest the WGRP meal according to instructions and data from this product for that subject was therefore excluded from the statistical analysis.

## Results

### Blood glucose responses

**Figure 1:**
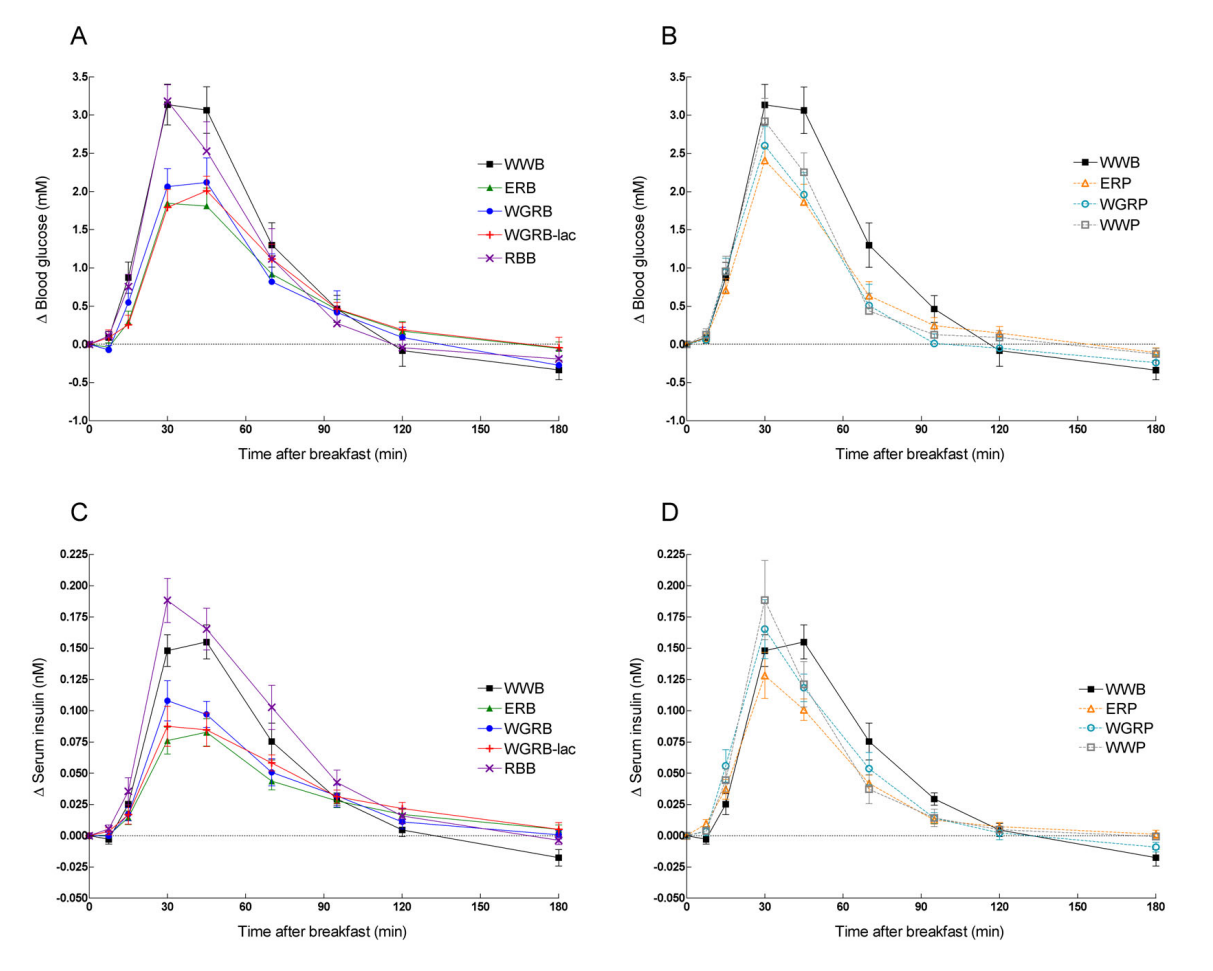
**Incremental change (Δ) in blood glucose in breads (A) and porridges (B).** Incremental change (Δ) in serum insulin in breads (C) and porridges (D). WWB is visible in all graphs. Values are means ± SEM, n = 11 for blood glucose and n = 12 for serum insulin (WGRP: n = 10 resp. 11). Significant treatment effect and time × treatment interactions were found, p < 0.0001 for both blood glucose and serum insulin (PROC MIXED in SAS).

The endosperm products ERB and ERP, as well as the whole grain products WGRB-lac and WGRP induced significantly lower incremental areas (iAUC 0-120 min) than WWB, with glycaemic indices (GI's) of 64, 70, 74 and 72, respectively (Table 4, Figure 1A, B). In the case of the ERB, WGRB and WGRB-lac, the early incremental blood glucose area (iAUC 0-30) was significantly reduced compared with WWB, WWP and RBB, respectively. When comparing the endosperm products ERP and ERB, a significantly larger incremental area was obtained with the porridge ERP in the early post-prandial phase (iAUC 0-30 min) (+51%) (Paired analysis ANOVA, p < 0.05, data not shown). Similarly, when comparing the whole grain products, WGRP and WGRB, respectively, the 30 min incremental area (iAUC 0-30 min) was 43% larger following the porridge (Paired analysis ANOVA, p < 0.05, data not shown).

**Table 4:** Blood glucose responses after the test meals

**Meals**	**GP**	**GPI**	**Glucose** **iAUC (0-30 min)**	**Glucose** **iAUC (0-120 min)**	**GI**
	*min/mM*	*%*	*min·mM*	*min·mM*	%
WWB	37.0 ± 5.6 ^b^	100 ± 0.0 ^b^	34.6 ± 4.1 ^a^	167.5 ± 17.8 ^a^	100 ± 0.0 ^a^
WWP	35.2 ± 5.0 ^b^	107 ± 15.6 ^b^	34.2 ± 4.7 ^a^	119.0 ± 13.0 ^ab^	77 ± 9.8 ^ab^
ERB	69.2 ± 10.1 ^a^	200 ± 24.9 ^a^	17.0 ± 3.2 ^c^	104.0 ± 15.9 ^b^	64 ± 7.5 ^b^
ERP	49.7 ± 6.3 ^ab^	145 ± 18.6 ^ab^	25.7 ± 3.0 ^abc^	103.1 ± 7.6 ^b^	70 ± 6.3 ^b^
WGRB	51.0 ± 7.0 ^ab^	142 ± 11.5 ^ab^	22.1 ± 3.6 ^bc^	118.9 ± 21.8 ^ab^	71 ± 9.7 ^ab^
WGRB-lac	74.3 ± 9.7 ^a^	226 ± 32.9 ^a^	17.9 ± 3.1 ^c^	113.6 ± 11.0 ^b^	74 ± 9.5 ^b^
WGRP	39.7 ± 7.3 ^b^	111 ± 17.7 ^b^	31.5 ± 3.8 ^ab^	110.0 ± 14.4 ^b^	72 ± 10.2 ^b^
RBB	35.7 ± 3.4 ^b^	113 ± 17.7 ^b^	33.5 ± 3.0 ^a^	147.2 ± 23.1 ^ab^	87 ± 6.7 ^ab^

Values are means ± SEM, n = 11 (WGRP: n = 10). Products not sharing the same letters were significantly different, p < 0.05 (ANOVA, followed by Tukey's test).

The glycaemic profile (GP, min/mM) was significantly higher for ERB and WGRB-lac compared with WWB, RBB, WWP and WGRP.

Significant differences in blood glucose were observed at specific time points (time × treatment p < 0.0001). RBB and WWB induced significantly higher glucose response than the non-supplemented rye products at 30 min (data not shown).

### Serum insulin responses

With the exception of RBB, all rye breads, and the ERP, induced significantly lower incremental insulin areas (iAUC 0-120 min) than WWB, resulting in insulinaemic Indices (II's) ranging from 61 to 73 (Table 5, Figure 1C, D). Instead, RBB showed an II of 128, with a significantly larger incremental area (iAUC 0-120 min) than all other rye products and the WWP. The insulin areas at 30 min (iAUC 0-30) were significantly smaller with the ERB, WGRB and WGRB-lac than with the enriched rye product RBB, and the porridges WGRP and WWP. Both rye porridges, ERP and WGRP, induced significantly higher insulin responses (iAUC 0-30 min) than the corresponding bread products, amounting to +96% and +87%, respectively. RBB and WWP induced significantly higher insulin incremental peaks compared to the ERB, WGRB and WGRB-lac

**Table 5:** Serum insulin responses after the test meals

**Meals**	**Insulin** **incremental peak**	**Insulin** **iAUC (0-30 min)**	**Insulin** **iAUC (0-120 min)**	**II**
	*nM*	*min·nM*	*min·nM*	%
WWB	0.168 ± 0.011 ^ab^	1.42 ± 0.15 ^ab^	8.35 ± 0.50 ^ab^	100 ± 0.0^ab^
WWP	0.201 ± 0.029 ^a^	1.96 ± 0.32 ^a^	7.19 ± 0.66 ^bc^	87 ± 7.8 ^bc^
ERB	0.089 ± 0.011 ^d^	0.76 ± 0.12 ^c^	4.99 ± 0.57 ^d^	61 ± 8.1 ^d^
ERP	0.131 ± 0.018 ^bc^	1.49 ± 0.24 ^ab^	5.77 ± 0.55 ^cd^	71 ± 6.9 ^cd^
WGRB	0.124 ± 0.013 ^bcd^	1.03 ± 0.16 ^bc^	6.06 ± 0.59 ^cd^	73 ± 7.5 ^cd^
WGRB-lac	0.103 ± 0.014 ^cd^	0.91 ± 0.20 ^bc^	5.98 ± 0.70 ^cd^	71 ± 8.9 ^cd^
WGRP	0.177 ± 0.019 ^ab^	1.93 ± 0.31 ^a^	7.31 ± 0.69 ^bcd^	88 ± 8.7 ^bcd^
RBB	0.202 ± 0.016 ^a^	1.87 ± 0.22 ^a^	10.45 ± 1.06 ^a^	128 ± 15.9^ a^

Values are means ± SEM, n = 12 (WGRP: n = 11). Products not sharing the same letters were significantly different, p < 0.05 (ANOVA, followed by Tukey's test).

Significant differences in serum insulin were observed at specific time points (time × treatment p < 0.0001). RBB induced significantly higher insulin response than all other rye products at 45 and 70 min (data not shown).

### Ghrelin responses

**Figure 2:**
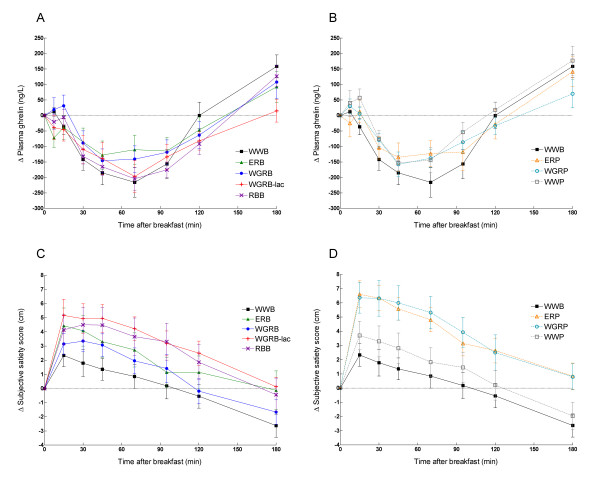
**Incremental change (Δ) in plasma ghrelin in breads (A) and porridges (B).** Incremental change (Δ) in subjective satiety scores in breads (C) and porridges (D). WWB is visible in all graphs. Values are means ± SEM, n = 12 (WGRP: n = 11). A significant treatment effect was found (p < 0.0001), but no significant time × treatment interaction, p = 0.67 for ghrelin and p = 0.97 for subjective feeling of satiety) (PROC MIXED in SAS)

Plasma ghrelin levels after all products decreased to a nadir, occurring at 64.9 ± 3.1 min in the post-prandial phase. (Table 6, Figure 2A, B). The ghrelin levels following WWB, ERP, WWP and RBB all rose significantly above the fasting level at 180 min, with the WWB and WWP causing a higher relative increase in ghrelin from fasting level to 180 min than WGRB-lac.

**Table 6:** Plasma ghrelin responses and subjective satiety responses after the test meals

**Meals**	**Ghrelin** **relative decrease (from fasting to nadir)**	**Ghrelin** **relative increase (0 to180 min)**	**Subjective satiety iAUC (0-180 min)**
	*%*	*%*	*min·cm*
WWB	-20.5 ± 2.4 ^a ^*	17.1 ± 4.6 ^a ^*	220 ± 71 ^c^
WWP	-17.2 ± 1.9 ^a ^*	15.5 ± 4.7 ^a ^*	371 ± 78 ^abc^
ERB	-16.7 ± 2.9 ^a ^*	8.0 ± 4.3 ^ab^	465 ± 146 ^abc^
ERP	-16.9 ± 3.6 ^a ^*	14.4 ± 5.2 ^ab ^*	716 ± 109 ^a^
WGRB	-16.9 ± 2. 8 ^a ^*	11.1 ± 5.4^ ab^	321 ± 99 ^bc^
WGRB-lac	-18.1 ± 3.5 ^a ^*	1.3 ± 3.4^ b^	570 ± 126 ^abc^
WGRP	-18.3 ± 2.1 ^a ^*	7.0 ± 4.0 ^ab^	718 ± 162 ^a^
RBB	-20.3 ± 1.8 ^a ^*	12.2 ± 4.0 ^ab ^*	587 ± 154 ^ab^

Values are means ± SEM, n = 12 (WGRP: n = 11). Products not sharing the same letters were significantly different, p < 0.05 (ANOVA, followed by Tukey's test). * indicates significant difference from fasting concentration (p < 0.05, ANOVA, followed by Tukey's test).

### Subjective satiety

WGRP and ERP induced significantly higher subjective satiety (expressed as iAUC from 0 to 180 min) than WWB and WGRB, with the iAUC for WGRP being 123% larger than that of WGRB (Table 6, Figure 2C, D). RBB induced a higher feeling of subjective satiety (iAUC) than WWB at 0-180 min.

**Table 7:** Correlations between blood glucose, serum insulin, plasma ghrelin and subjective satiety responses following the test meals

	**GP**	**II**	**Insulin** **Incremental peak**	**GI**	**Hypoglycaemia** **neg AUC (30-180 min)**
**Subjective satiety** 180 min (delta from fasting)	NS	NS	NS	NS	-0.28*

**Ghrelin** relative increase (0 to180 min)	-0.40 ***	0.33 **	0.34 **	NS	0.26*

**Hypoglycaemia** neg AUC ^1^ (30-180 min)	-0.43 ***	0.22 *	0.38 ***	-0.39 ***	
	
**GI**	NS	0.35 **	NS		
		
**Insulin** incremental peak	-0.64 ***	0.72 ***			
			
**II**	-0.48 ***				

* p < 0.05, ** p < 0.01, *** p < 0.001, NS = non significant. (Spearman's partial coefficients controlling for subjects (two-tailed test)).

### In vitro rate of starch hydrolysis

All porridges and the ERB were characterized by a lower hydrolysis index (HI) than WWB and WGRB. In addition, WGRP showed a lower HI than WGRB-lac, RBB and ERP (Table 1).

### Correlations

Correlations between II, insulin incremental peak, GI, GP, hypoglycaemia, ghrelin and subjective satiety are presented in Table 7. The hydrolysis index (HI) was neither correlated with the GI, II, GP or with the hypoglycaemia. The subjective satiety (iAUC 0-180 min) was not correlated with the protein, caloric, starch, fat, water or fibre contents of the test meals.

## Discussion

In the present study, the endosperm products and whole grain rye breads induced significantly lower II's than WWB, which is in agreement with previous findings [24-26]. In contrast to the other rye breads in the study, the RBB i.e. white wheat flour enriched with rye bran (35 wt %) showed a significantly higher II compared to all other rye products.

To explain the low post-prandial insulin response of the endosperm and whole grain rye products, the course of glycaemia was analyzed. The rye products tended to induce blood glucose curves that remained above fasting for a longer time, with a lower glucose peak and a less pronounced late hypoglycaemia. It could be hypothesized that the inconsistency between GI and II, reported for some rye products, is caused by this low but prolonged net increment in post-prandial blood glucose response, resulting in an improved insulin economy, but maintaining a high GI as calculated from the 120 min area. In order to quantify the profile of the blood glucose curve, the glycaemic profile (GP) was introduced, defined as the duration for incremental post-prandial blood glucose response divided with the blood glucose incremental peak. Thus, a high GP is indicative of a facilitated post-prandial glycaemic regulation, with a lower glucose peak and a less pronounced hypoglycaemia. As judged from their higher GP's, it could be argued that ERB and WGRB-lac are characterized by a more beneficial glucose regulation than RBB, WWB, WWP and WGRP, respectively.

In the present study, the II showed a stronger correlation with GP than with the GI of the products. Furthermore, the insulin incremental peak was negatively correlated to the GP but showed no correlation with the GI. This indicates that the GP is a better predictor, than the GI, of the acute insulin response of rye products.

We suggest that the GP is a useful tool for evaluation of post-prandial glycaemia to carbohydrate foods in general. Granfeldt et al. [31] noted that although the time course for the post-prandial glycaemia were considerably different with pasta and white wheat bread in healthy elderly subjects; the GI's remained similar due to enduring incremental blood glucose response in the late phase with pasta. If calculating GP values from estimated data in that study; GP's of 120 and 41 were estimated for pasta and bread, respectively. The high GP pasta meal significantly improved glucose tolerance at a standardized "second-meal", ingested after 4 h, compared with the low GP white wheat bread reference [32]. Moreover, when studying a range of cereal breakfasts in healthy subjects it was found that the blood glucose level 4 h after commencing the test breakfast was negatively correlated to the blood glucose incremental peak at a following standardized lunch (r = -0.29, p = 0.043) [33]. These results suggest that products characterized by high GP's are more prone to induce benefits on second-meal glucose tolerance.

The beneficial glycaemic profile and low post-prandial insulin response seen with endosperm rye bread does not rule out a dietary fibre-related mechanism. In contrast to WWB (1.8 g DF/100 g bread), the endosperm rye bread (ERB) are rich in soluble fibres (6.7 g DF/100 g bread of which 2.5 g was soluble and 4.2 g insoluble fibres). However, since the more fibre rich RBB appeared to be devoid of acute metabolic benefits, it could be suggested that some unknown property or component(s) present in the endosperm, but not the bran fraction of rye, affects the course of glycaemia and lowers the insulin demand. This is a new observation showing that the benefits of rye products on insulin demand cannot be mimicked by adding rye bran to a white wheat background. The results also show the importance of maintaining all parts of the whole grain, including the endosperm, in rye products. The improved insulin economy seen with endosperm and whole grain rye breads could possibly be due to an improvement in insulin sensitivity or by an increase in first phase insulin release, previously seen with rye breads [34,35].

Whole grain and endosperm rye porridges induced significantly higher early insulin and glucose responses (0-30 min) compared with the corresponding bread products. Also the insulin incremental peak was higher for the porridges; ERP inducing a 48% higher peak than ERB and WGRP a 42% higher insulin peak than WGRB. Thus, in the present study, the type of processing affects the glycaemic and hormonal responses to flour based rye products, in favour of bread making.

In a study by Juntunen et al [25] it was suggested that a lowered post-prandial insulin response to rye products could be explained by a mechanically firmer structure, leading to obstructed amylolysis and a slower rate of glucose delivery. The hydrolysis index (HI), calculated with WWB as reference, was lower than that of WWB for some, but not all whole grain and endosperm rye products in the present study. HI was not correlated with GI, GP or II. Hence, in the present work, the high GP and low II of rye products are not explained by obstructed amylolysis.

A connection between low II features of foods and increased post-meal satiety has been shown in several studies comparing equicarbohydrate food portions [36-39]. In the present study, the insulin incremental peak was related to the extent of hypoglycaemia (neg AUC 30-180); a higher insulin surge being related to a more pronounced dip in blood glucose below fasting level (r = 0.38, p < 0.01). The extent of hypoglycaemia was in turn negatively correlated to the feeling of subjective satiety at 180 min (r = -0.28, p < 0.05). As a possible measure of appetite regulation, we studied total plasma ghrelin in the post-prandial phase. In the present study, a high insulin incremental peak was related to a more potent recovery of ghrelin in the later post-prandial phase (r = 0.34, p < 0.01). Also, previous studies, both clamp [40-42] and meal studies [43,44], has found that total ghrelin concentrations were influenced by insulin. High levels of total ghrelin at 4 h after a preload has been demonstrated to increase voluntary energy intake at a subsequent meal [45]. Thus, it can be hypothesized that a whole grain- or endosperm rye bread breakfast, causing low acute insulin response might reduce hunger in the late post-prandial phase and possibly lower energy intake at a subsequent meal compared with a high II breakfast such as WWB. Semi-acute and longer term studies are needed to verify this hypothesis.

## Conclusion

Endosperm rye products and whole grain rye breads induced significantly lower II's than white wheat bread (WWB). In addition, these products induced low and prolonged glucose profiles i.e. high GP's, in the post-prandial phase. The rye bran bread, devoid of the endosperm part of the rye grain, induced GI, GP and II similar to that of a WWB. The finding that the presently introduced GP better predict the insulin response of rye products in the acute post-prandial phase, compared with the GI, is important. It is put forward that the GP could be exploited when evaluating post-prandial glycaemia of food products. The results also indicate that a higher acute insulin response was associated with more prominent late hypoglycaemia, feeling of hunger and an increase in plasma ghrelin, respectively. Thus, low II rye breakfast products may improve appetite regulation. The latter warrants further investigations which are currently under way.

## List of abbreviations

BMI: body mass index; CVD: cardiovascular diseases; ERB: endosperm rye bread; ERP: endosperm rye porridge; GI: glycaemic index; GP: Glycaemic profile; HI: hydrolysis index; iAUC: incremental area under the curve; II: insulinaemic index; neg AUC: negative area under the curve; RBB: rye bran bread; WGRB: whole grain rye bread; WGRB-lac; whole grain rye bread made with lactic acid; WGRP: whole grain porridge; WWB: white wheat bread; WWP: white wheat porridge.

## Competing interests

The authors declare that they have no competing interests.

## Authors' contributions

LAHR coordinated the study and was responsible for the study design, the collection and analysis of the data, statistical analysis and for writing the paper. EMÖ was involved in the study design, interpretation of data and in writing the paper. IMEB was the guarantor for the founding of the study and was involved in the study design, interpretation of data and writing of the paper. LOBS was involved in the analysis and statistical analysis of total ghrelin. UKA and CH was involved in the study design.
